# Clinical Presentation and Surgical Management of Penile Carcinoma: A Retrospective Case Series from a Tertiary Referral Center

**DOI:** 10.3390/diagnostics16172854

**Published:** 2026-09-05

**Authors:** Arsenie Dan Spînu, Lucia Bubulac, Radu Andrei Amza, Adrian Constantin, Luciana Alexandra Pavelescu, Ştefan Milicescu, George Ion, Daniela Miricescu, Ana Maria Alexandra Stanescu, Viorica Tudor

**Affiliations:** 1 Department 3, Faculty of Medicine, “Carol Davila” University of Medicine and Pharmacy, 020021 Bucharest, Romania; arsenie.spinu@umfcd.ro (A.D.S.); radu-andrei.amza@drd.umfcd.ro (R.A.A.); 2 Urology Department, “Dr. Carol Davila” Central Military Emergency University y Hospital, 010825 Bucharest, Romania; 3 Department of Family Medicine III, Faculty of Medicine, “Carol Davila” University of Medicine and Pharmacy, 020021 Bucharest, Romania; 4 General and Esophageal Surgery Discipline, Faculty of Medicine, “Carol Davila” University of Medicine and Pharmacy, 020021 Bucharest, Romania; adrian.constantin@umfcd.ro; 5 Surgery Department, St. Mary’s Clinical Hospital, 011192 Bucharest, Romania; 6 Cellular, Molecular Biology and Histology Discipline, “Carol Davila” University of Medicine and Pharmacy, 050474 Bucharest, Romania; luciana.pavelescu@umfcd.ro; 7 Aesthetics in Dental Medicine Department, Faculty of Dentistry, “Carol Davila” University of Medicine and Pharmacy, 020021 Bucharest, Romania; stefan.milicescu@umfcd.ro; 8 Fixed Dental Prosthetics and Occlusology Department, Faculty of Dentistry, “Carol Davila” University of Medicine and Pharmacy, 020021 Bucharest, Romania; george_ion@umfcd.ro; 9 Biochemistry Department, Faculty of Dentistry, “Carol Davila” University of Medicine and Pharmacy, 020021 Bucharest, Romania; 10 Department I of Family Medicine and Clinical Base, “Dr. Carol Davila” Central Military Emergency University Hospital, 010825 Bucharest, Romania; alexandra.stanescu@umfcd.ro; 11 Integrated Outpatient Department, “Prof. Dr. Agrippa Ionescu” Clinical Emergency Hospital, 011356 Bucharest, Romania; tudorviorica_med@yahoo.com

**Keywords:** penile carcinoma, squamous cell carcinoma, HPV-associated carcinoma, immunohistochemistry, p16, lymph node metastasis, surgical management, prognosis, retrospective case series, PET/CT

## Abstract

**Background and Clinical Significance:** Penile carcinoma is a rare malignancy in developed countries, yet it remains associated with significant morbidity due to its aggressive local behavior and potential for lymphatic dissemination. Human Papillomavirus (HPV) infection plays a central role in its pathogenesis, while lymph node involvement represents the most important prognostic factor. **Case Presentation:** In this retrospective case series (*n* = 3), we evaluated three representative patients treated at a tertiary referral center in Romania between August 2020 and December 2024. Clinical, pathological, and therapeutic data were analyzed, with emphasis on tumor stage, nodal involvement, surgical management, and follow-up outcomes. Immunohistochemical analysis, including p16 expression, was performed to assess HPV-related tumor profiles. **Conclusions:** Three representative cases illustrating distinct clinical scenarios were included. Patients with localized disease and negative lymph nodes achieved favorable long-term outcomes, including 3-year disease-free survival (documented follow-up through December 2023; follow-up data beyond 2023 were unavailable, capping documented disease-free follow-up at 36 months). In contrast, advanced disease with nodal metastasis was associated with poor prognosis despite aggressive multimodal treatment. Immunohistochemical profiling revealed both HPV-associated and HPV-independent tumor patterns, which appeared to correlate with clinical outcomes. A case of locally advanced disease demonstrated that radical surgical intervention combined with adjuvant therapy may achieve prolonged recurrence-free survival. Penile carcinoma requires prompt diagnosis and individualized management.

## 1. Introduction

Penile carcinoma is a rare but clinically significant malignancy, accounting for less than 1% of all male cancers in developed countries [1,2]. Despite its low incidence, the disease is associated with considerable morbidity and mortality due to its locally aggressive behavior, potential for regional and distant metastasis, and profound impact on patients’ quality of life [3]. Beyond the oncological burden, penile cancer carries important functional and psychological consequences, often affecting body image, sexual function, and social integration [4]. In this context, early recognition and appropriate management are essential for improving both survival outcomes and long-term clinical and functional results.

The pathogenesis of penile carcinoma is complex and multifactorial, with infection by high-risk Human Papillomavirus (HPV), particularly genotypes 16 and 18, playing a central etiological role [5]. Persistent HPV infection drives malignant transformation by integrating viral DNA into host epithelial cells and dysregulating key tumor suppressor pathways [6]. In routine clinical practice, p16 immunohistochemical expression is widely used as a surrogate marker for oncogenic HPV infection and has been identified in a significant proportion of penile squamous cell carcinomas (penile SCC) [7]. In addition to viral factors, several well-established risk factors contribute to disease development, including lack of circumcision, phimosis, chronic inflammation, poor genital hygiene, smoking, and high-risk sexual behavior [8].

From a clinical perspective, penile carcinoma most often presents as a visible lesion that may be nodular, ulcerative, or exophytic [9]. However, diagnosis is often delayed, particularly in patients with phimosis, in whom lesions may remain concealed beneath the foreskin. As a result, patients may present with locally advanced disease at the time of diagnosis [10]. A thorough clinical examination, including careful inspection and palpation of the entire penile structure and the inguinal regions, is essential for accurate assessment of tumor extent and regional lymph node involvement [11].

Among all prognostic factors, lymph node status remains the most important determinant of survival. Penile carcinoma typically spreads via the lymphatic system in a stepwise manner, initially involving the inguinal lymph nodes and subsequently the pelvic nodal stations [11]. The presence and extent of nodal metastasis have a dramatic impact on outcomes, with five-year cancer-specific survival rates decreasing from approximately 90–95% in node-negative disease to less than 40% in patients with advanced nodal involvement [12]. These findings underscore the critical importance of accurate staging and timely therapeutic intervention.

The management of penile carcinoma is primarily surgical and requires an individualized approach based on tumor stage, anatomical involvement, and patient-related factors [13]. Organ-sparing techniques may be considered in selected early-stage cases; however, partial or total penectomy remains necessary in more advanced disease to achieve oncological control [14]. Inguinal lymph node dissection serves both as a staging procedure and as a therapeutic intervention. In patients with high-risk features or advanced nodal disease, multimodal treatment strategies incorporating radiotherapy and chemotherapy may improve outcomes, although they are associated with increased morbidity [15]. Furthermore, long-term survivors frequently experience complications such as lymphedema, urethral strictures, and impaired sexual function, underscoring the need for comprehensive postoperative care and follow-up [16].

Given the rarity of penile carcinoma, much of the current knowledge is based on limited case series and retrospective analyses. In this context, detailed clinical observations remain valuable for improving understanding of disease behavior and for optimizing management strategies.

Therefore, the aim of the present study is not only to describe the clinical and surgical management of penile carcinoma through a series of representative cases treated at a tertiary referral center, but also to highlight the direct correlation among tumor stage, lymph node involvement, surgical strategy, and long-term outcomes in real-world clinical practice. By illustrating distinct clinical scenarios ranging from early-stage disease to advanced locoregional involvement, this study provides a clinically oriented perspective on decision-making and prognostic stratification. Furthermore, by integrating these findings with current literature, we aim to emphasize the practical implications of timely diagnosis, appropriate surgical aggressiveness, and multimodal treatment approaches in optimizing patient survival and quality of life.

## 2. Case Presentation

### 2.1. Study Design and Participants

This retrospective case series was designed to explore clinicopathological and molecular correlations in penile SCC. The study was conducted at the Department of Urology of the “Dr. Carol Davila” Central Military Hospital, a tertiary referral center in Bucharest, Romania.

Patient data (*n* = 3) were collected and analyzed qualitatively from August 2020 to December 2024. Given the rarity of penile carcinoma, a case-based approach was used to provide a detailed clinical perspective on disease evolution and management in real-world practice.

### 2.2. Patient Selection

Patients were identified through the institutional electronic medical database based on a confirmed diagnosis of penile malignancy.

The inclusion criteria were as follows:

- age ≥ 18 years;
- histopathological confirmation of penile squamous cell carcinoma;
- surgical treatment performed at our institution (including penectomy and/or lymphadenectomy);
- availability of complete clinical, imaging, and pathological records;
- documented postoperative follow-up.

### 2.3. Exclusion Criteria

Patients were excluded if they met any of the following criteria:

- incomplete clinical or follow-up data;
- presence of synchronous urogenital malignancies;
- non-squamous histological subtype.

### 2.4. Clinical and Pathological Assessment

All patients underwent a comprehensive clinical evaluation, including detailed inspection and palpation of the penile lesion and regional lymph nodes. Tumor characteristics, including size, anatomical location, and degree of local invasion, were recorded.

Staging followed the European Association of Urology (EAU) guidelines. Imaging studies, primarily computed tomography (CT), were used to assess regional lymph node involvement and to exclude distant metastasis.

Histopathological examination confirmed the diagnosis and provided information regarding tumor differentiation and invasion patterns.

In addition to standard histopathological examination, we performed immunohistochemical (IHC) analysis in all cases to further characterize tumor biology. The panel included p16 (as a surrogate marker for HPV infection), p53, p63, Ki-67, and cytokeratin AE1/AE3. These markers were used to assess tumor proliferation, differentiation, and HPV-related oncogenic pathways.

### 2.5. Surgical Management and Multimodal Treatment

Treatment strategies were individualized based on tumor stage, anatomical involvement, and patient-related factors.

The surgical procedures performed included:

- partial or total penectomy, aiming to achieve oncological control with negative resection margins;
- inguinal lymph node dissection, performed for both staging and therapeutic purposes in patients with clinically suspected or confirmed nodal involvement;
- radical surgery (total emasculation with perineal urethrostomy) in cases of locally advanced disease involving the penile root or corpora cavernosa.

In selected cases with high-risk pathological features or advanced nodal disease, adjuvant therapy (radiotherapy and/or chemotherapy) was administered as part of a multimodal treatment approach.

### 2.6. Follow-Up and Outcome Measures

Postoperative follow-up consisted of periodic clinical evaluations and imaging studies (CT scans), in accordance with institutional protocols.

The main outcomes assessed included:

- cancer-specific survival, correlated with nodal status;
- recurrence-free survival, defined as the absence of clinical or radiological evidence of disease recurrence during follow-up;
- treatment-related complications, including lymphedema, urethral strictures, and radiation-induced adverse effects.

### 2.7. Data Analysis

Given the limited number of cases, a descriptive analytical approach was used. Clinical, pathological, and treatment-related variables were analyzed qualitatively, with emphasis on the correlation among tumor stage, lymph node involvement, therapeutic strategy, and clinical outcomes.

### 2.8. Ethical Considerations

Ethical approval for this retrospective study was granted by the Institutional Review Board of the Central Military Hospital “Dr. Carol Davila”, Bucharest, Romania (protocol code no. 849/21.01.2026; approval date: 21 January 2026).

Written informed consent for surgical procedures and institutional research or publication use of anonymized clinical data was routinely obtained from all patients at the time of admission.

Preoperative staging pelvic cross-sectional imaging (contrast-enhanced CT) was performed before the primary surgery and showed no lymph node enlargement or pathological suspicion of pelvic lymph nodes (cN0). The occurrence of pelvic recurrence at 3 months postoperatively strongly suggests undetected baseline occult microscopic pelvic disease rather than late de novo progression.

### 2.9. Case 1

A 67-year-old male with a medical history of chronic heart failure, arterial hypertension, and prior gastric ulcer surgery was diagnosed with penile carcinoma. The patient underwent partial penectomy combined with bilateral superficial and deep inguinal lymph node dissection.

Preoperative staging pelvic cross-sectional imaging (contrast-enhanced CT) was performed prior to the primary surgical procedure and showed no evidence of pathological lymphadenopathy in the deep inguinal or pelvic regions (cN0). The occurrence of pelvic disease progression or recurrence at 3 months postoperatively strongly indicates the presence of undetected baseline occult microscopic pelvic nodal involvement rather than late de novo progression.

Histopathological examination revealed metastatic involvement of the right inguinal lymph nodes. Three months after the initial intervention, locoregional disease progression was identified, necessitating a second surgical procedure consisting of extended ilio-obturator lymphadenectomy. Histopathological analysis confirmed persistent nodal metastases.

Postoperatively, the patient received adjuvant chemotherapy and radiotherapy. Despite multimodal treatment, the disease progressed, and the patient died 31 months after the initial surgery.

Immunohistochemical analysis showed negative p16 and p53 expression, with positive p63 staining in tumor cells. Ki-67 expression was predominantly in the lower third of the squamous epithelium, indicating a moderate proliferative index. These findings were consistent with a well-differentiated squamous cell carcinoma with an HPV-independent molecular profile.

**Figure 1:**
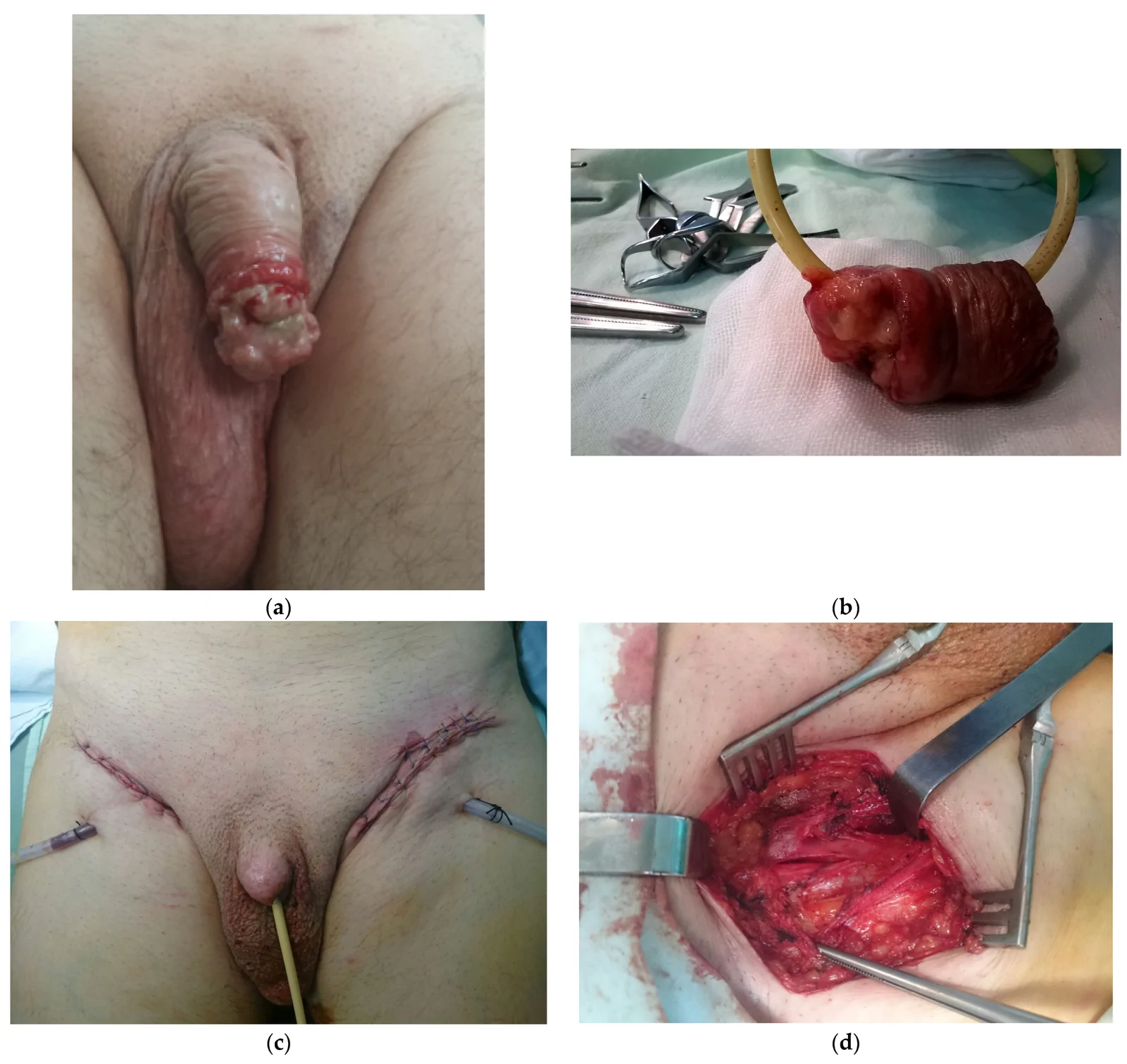
Case 1 (pT2pN2, Grade 2, p16-negative/HPV-independent): (**a**) Preoperative view showing exophytic tumor growth; (**b**) Macroscopic surgical specimen following partial penectomy; (**c**) Final postoperative appearance after partial penectomy with superficial and deep inguinal lymph node dissection; (**d**) Intraoperative view of superficial and deep inguinal lymph node dissection.

The clinical presentation, intraoperative findings, and surgical management are shown in Figure 1.

### 2.10. Case 2

A 53-year-old male with a history of hiatal hernia, gastroesophageal reflux disease, and prior appendectomy presented with a penile mass. Clinical and imaging evaluation demonstrated a tumor predominantly involving the corpora cavernosa, with limited extension to the glans and invasion toward the penile root, consistent with locally advanced disease.

The patient underwent total emasculation with perineal urethrostomy and bilateral superficial and deep inguinal lymph node dissection. Postoperative management included adjuvant chemotherapy and radiotherapy.

During follow-up, the patient showed no clinical or radiological evidence of recurrence and achieved a recurrence-free survival of approximately 3 years and 8 months.

Immunohistochemical analysis revealed negative p16 expression and strong p63 positivity in tumor cells. Ki-67 showed a high proliferative index of approximately 80%. The overall immunophenotypic profile supported the diagnosis of a well-differentiated squamous cell carcinoma with an HPV-independent molecular profile.

**Figure 2:**
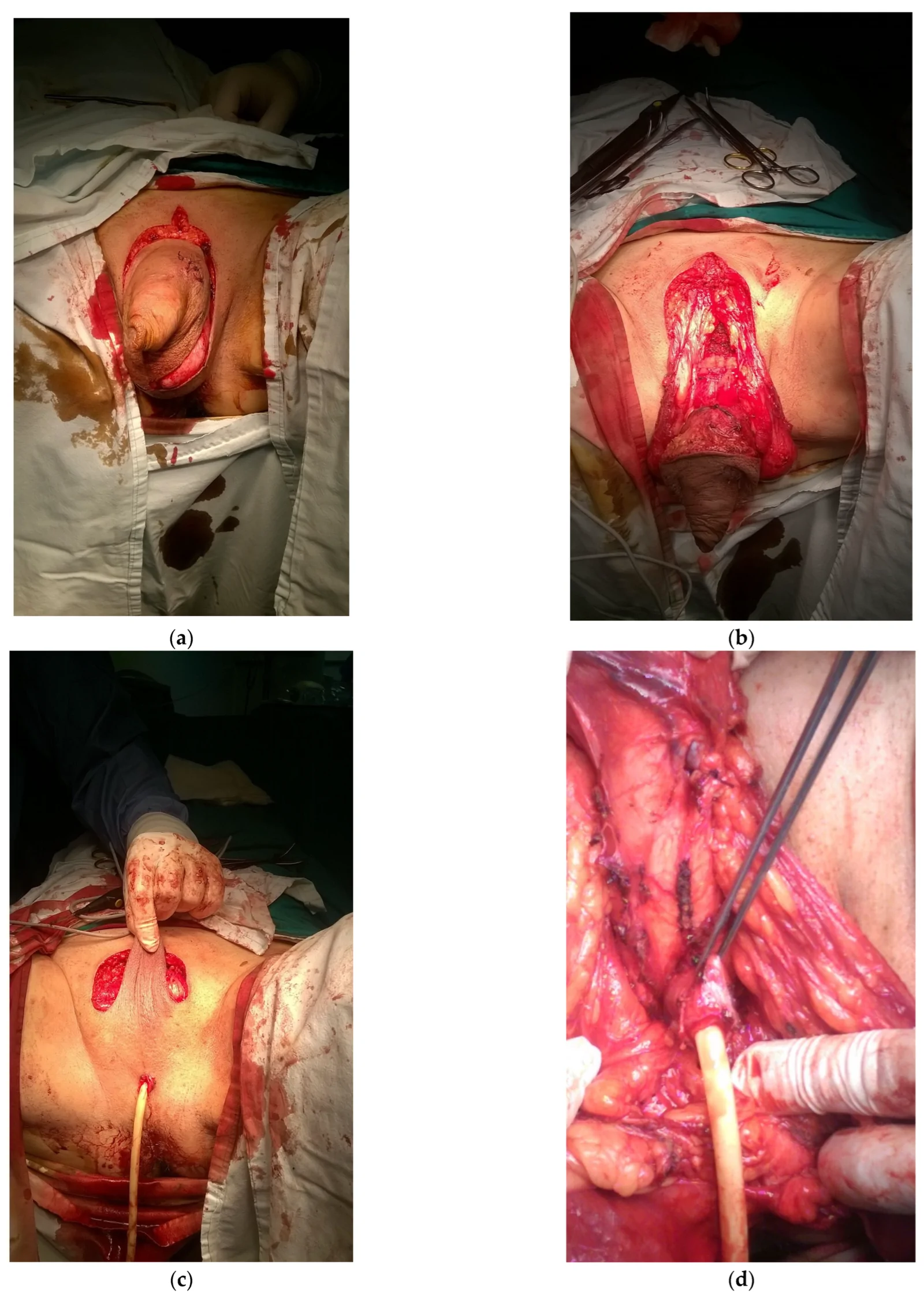
Case 2 (pT3pN3, Grade 3, p16-negative/HPV-independent): (**a**) Initial circumferential incision showing diffuse infiltration of the penile shaft; (**b**) Intraoperative view of radical total emasculation; (**c**) Urethral aspect prior to perineal urethrostomy; (**d**) Final perineal reconstruction with perineal urethrostomy.

The main surgical steps and reconstructive aspects of this case are shown in Figure 2.

### 2.11. Case 3

A 73-year-old male with a medical history of hypertension, chronic heart failure, and prior appendectomy presented with a suspicious penile lesion, which was histopathologically confirmed as penile carcinoma.

The patient underwent partial penectomy and bilateral superficial inguinal lymph node dissection. A superficial-only inguinal dissection was chosen for staging to minimize surgical morbidity while maintaining staging accuracy, given the patient’s low-risk primary tumor stage (well-differentiated pT1a) and significant cardiovascular comorbidities.

Staging lymphadenectomy revealed no nodal involvement (pN0, 0/14 nodes positive). However, microscopic examination demonstrated adverse features, including focal lymphovascular invasion (LVI) and close surgical margins (<2 mm). Following multidisciplinary tumor board evaluation, adjuvant radiotherapy was administered to the bilateral inguinal regions due to these high-risk adverse primary histological features and concern for occult microinvasion.

The patient achieved 3-year disease-free survival (documented follow-up through December 2023; follow-up data beyond December 2023 were unavailable, capping documented disease-free follow-up at 36 months).

**Figure 3:**
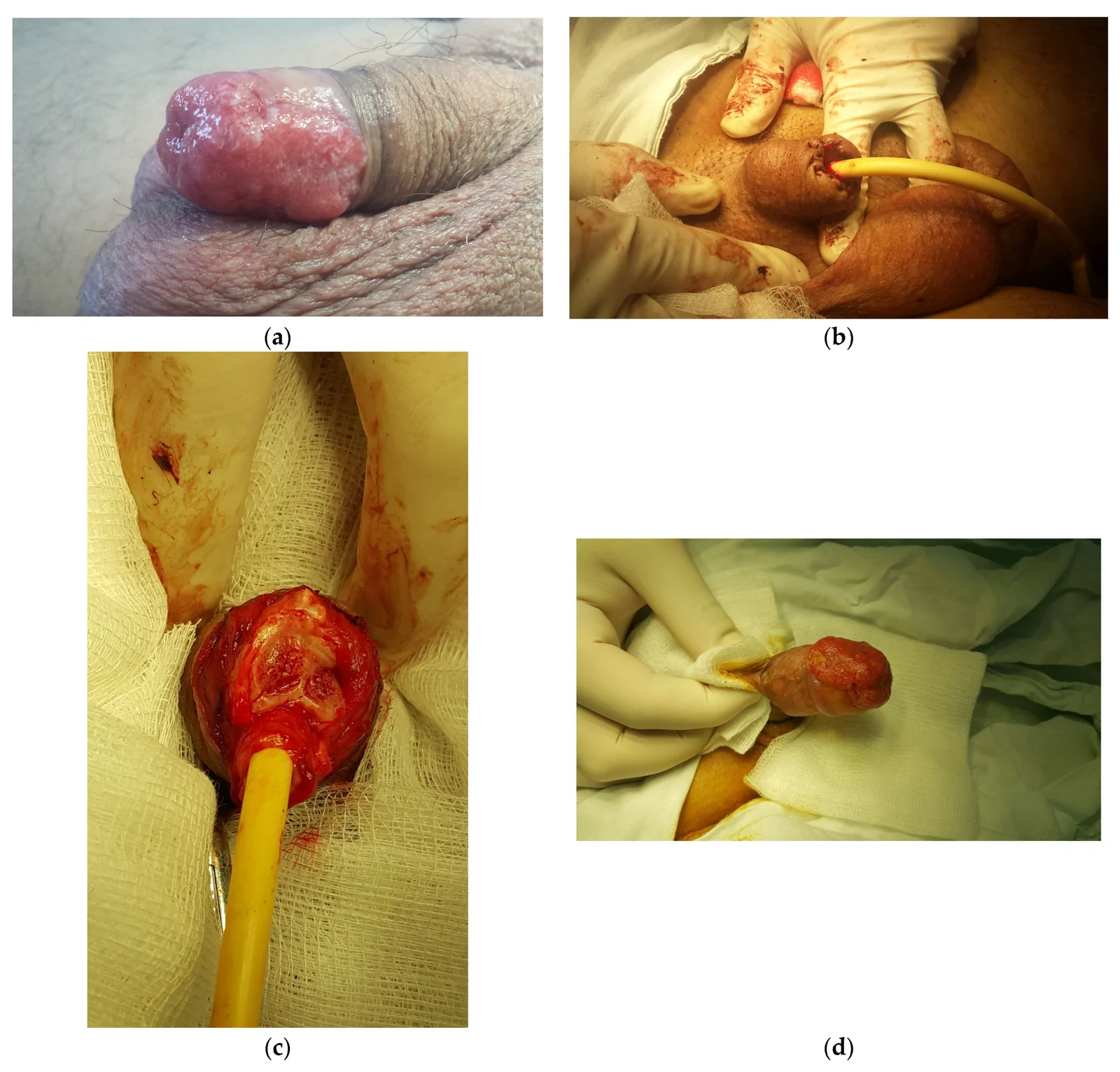
Case 3 (pT1aN0, Grade 1, p16-positive/HPV-associated): (**a**) Preoperative penile lesion; (**b**) Preoperative view of the tumor involving at least half of the glans; (**c**) Intraoperative view showing the corpora cavernosa and urethra with a Foley catheter; (**d**) Final postoperative appearance after partial penectomy and bilateral superficial inguinal dissection.

Preoperative, intraoperative, and postoperative findings are shown in Figure 3.

### 2.12. Summary of Clinical Characteristics, Treatment, and Outcomes

The key clinical features, treatment strategies, and outcomes of the three cases are summarized in Table 1. The data highlight variability in clinical presentation and prognosis, particularly regarding lymph node involvement and the extent of surgical treatment.

## 3. Discussion

Cancer is the leading global cause of mortality [17]. Therefore, early detection using various techniques and molecular evaluations significantly improves survival rates [18,19].

Penile carcinoma remains a rare malignancy in developed countries; however, its clinical relevance is disproportionately high because of its aggressive biological behavior, potential for lymphatic dissemination, and profound impact on patient quality of life [16]. This case series offers a clinically oriented perspective on disease heterogeneity, highlighting the interplay between tumor stage, lymph node involvement, therapeutic strategy, and patient outcomes in a real-world tertiary referral setting.

**Figure 4:**
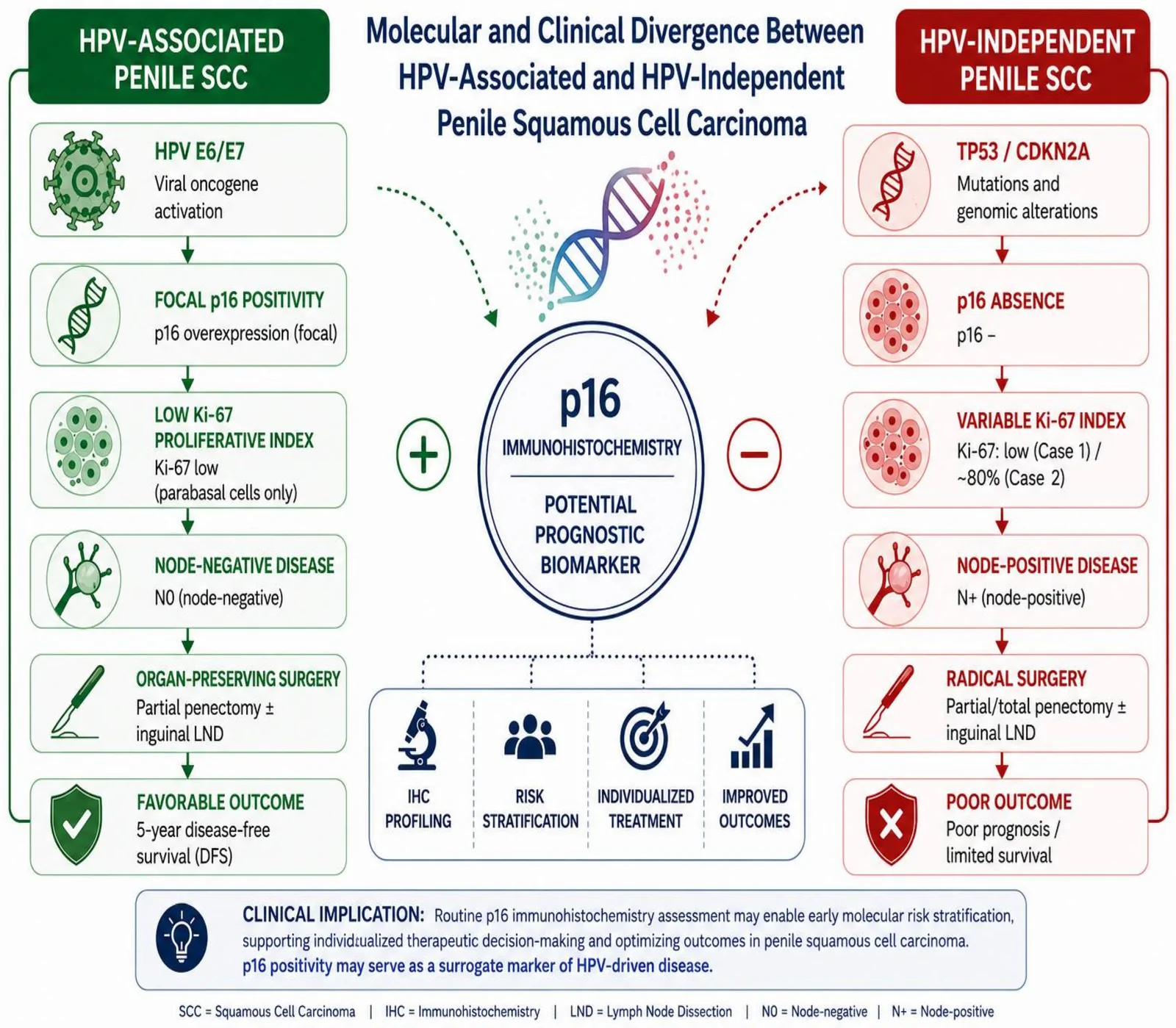
HPV-associated penile squamous cell carcinoma versus HPV-independent penile squamous cell carcinoma.

A central aspect in the contemporary understanding of penile carcinogenesis is the role of HPV, particularly high-risk genotypes such as HPV-16 and HPV-18 [20]. Epidemiological data indicate that approximately 30% of men worldwide are infected with HPV, with high-risk types present in a significant subset of cases. In the 2022 WHO classification, penile SCC is stratified by HPV status, reflecting distinct molecular pathways of tumorigenesis [21]. In this context, p16 immunohistochemistry has emerged as a reliable surrogate marker for HPV-driven oncogenesis, with reported positivity rates of approximately 40% in penile cancers and significantly higher expression in HPV-related tumors compared to HPV-negative counterparts [22]. Although molecular testing is not universally available, p16 remains a valuable and accessible tool in routine pathological assessment [23] (Figure 4).

In this context, an additional strength of the present case series is the incorporation of immunohistochemical profiling, which enabled the distinction between HPV-associated and HPV-independent molecular pathways. Two of the presented cases (Cases 1 and 2) exhibited an HPV-independent profile, characterized by negative p16 expression and variable proliferative indices, whereas Case 3 demonstrated features consistent with HPV-associated carcinogenesis.

Importantly, in our case series, we observed that p16-positive tumors were associated with more favorable short-term surgical outcomes than p16-negative advanced disease. Given our limited cohort of three patients, these findings are clinical observations rather than generalized prognostic conclusions. These observations align with current literature highlighting the biological heterogeneity of penile SCC and the potential prognostic relevance of HPV status.

A methodological limitation of this study is its exclusive reliance on p16 INK4a immunohistochemistry as a surrogate marker for HPV infection. Although p16 is widely accepted in clinical practice, it does not equate to direct viral identification. Testing for HPV DNA via PCR or E6/E7 mRNA transcripts was not performed in this series [24].

To fully capture contemporary diagnostic advances, the potential role of 18F-FDG PET/CT warrants consideration in penile cancer management. Conventional CT and MRI have limited sensitivity for detecting occult lymph node metastasis. 18F-FDG PET/CT offers superior accuracy for regional and distant staging, refining patient selection for invasive inguinal lymph node dissection, sparing node-negative patients from surgical morbidity, and identifying candidates for neoadjuvant therapy [24].

Beyond etiological considerations, the present study underscores the critical importance of early diagnosis. This aligns with observations in other aggressive malignancies, such as cutaneous melanoma, where prognosis is closely linked to early detection and accurate staging [25]. Penile carcinoma typically presents as a visible lesion; however, diagnostic delay remains a significant clinical challenge, particularly in patients with phimosis or limited access to specialized care [9]. This delay often leads to presentation at more advanced stages, as illustrated by the first and second cases included in this series. The heterogeneity of clinical presentation underscores the need for a thorough physical examination, including inspection and palpation of the entire penile structure and inguinal regions, as recommended by current EAU guidelines.

Among prognostic factors, lymph node involvement remains the most significant determinant of survival. Penile carcinoma follows a predictable pattern of lymphatic spread, initially involving the inguinal nodes and subsequently the pelvic nodal stations [26]. Large clinical series have shown a marked decline in five-year cancer-specific survival with increasing nodal burden, ranging from approximately 90–95% in node-negative disease to below 40% in patients with advanced nodal involvement [27,28]. The cases presented in this study reflect this well-established prognostic gradient. The first case, characterized by early nodal metastasis and rapid locoregional progression, resulted in poor survival despite aggressive multimodal treatment. In contrast, the third case, with no evidence of nodal involvement and negative surgical margins, demonstrated excellent long-term oncological control. These findings suggest a potential interaction between molecular profile and nodal status in shaping clinical outcomes.

The second case offers additional insight into the management of locally advanced disease. The tumor showed predominant invasion of the corpora cavernosa with limited glans involvement, a less common pattern of tumor extension that may contribute to diagnostic delay. In this context, radical surgical intervention in the form of total emasculation, combined with adjuvant chemoradiotherapy, achieved prolonged recurrence-free survival. This observation supports the role of aggressive multimodal treatment strategies in selected patients with advanced local disease, although such approaches must be carefully balanced against functional and psychological outcomes.

From a therapeutic perspective, surgery remains the cornerstone of penile cancer management. Achieving negative resection margins is essential for local disease control, and inguinal lymph node dissection serves both staging and treatment purposes [29]. Current guidelines emphasize a risk-adapted approach to lymph node management, with sentinel lymph node biopsy and selective lymphadenectomy increasingly used to reduce overtreatment [30]. However, in patients with clinically evident nodal disease, as observed in the first case, more extensive surgical intervention remains necessary.

Another important aspect highlighted by this study is the burden of treatment-related morbidity. Even among patients with favorable oncological outcomes, long-term complications such as lymphedema, urethral strictures, and radiation-induced tissue damage may significantly impact quality of life [31].

The third case illustrates this point, with late neo-urethral stenosis successfully managed surgically. These findings reinforce the need for long-term follow-up and multidisciplinary care. Overall, this case series reflects the challenges of managing penile carcinoma in a tertiary referral center, where patients may present at advanced stages due to delayed diagnosis or referral. At the same time, it highlights the importance of early recognition, accurate nodal staging, individualized surgical planning, and coordinated multidisciplinary management to optimize patient outcomes.

### 3.1. Clinical Implications for Practice

The findings of this case series offer several clinically relevant insights that may inform decision-making in managing penile carcinoma. Given the close relationship between lymph node involvement and prognosis, early and accurate nodal staging should be systematically incorporated into the initial evaluation. Timely inguinal lymph node assessment—whether through imaging, sentinel node techniques, or surgical dissection—plays an important role in both staging and therapeutic planning. In addition, the variability in clinical presentation underscores the need for a high index of suspicion, particularly in patients with risk factors such as phimosis or delayed presentation [32]. The cases presented also emphasize the importance of an individualized surgical approach that balances oncological control with functional preservation whenever feasible. While organ-sparing techniques may be appropriate in early-stage disease, advanced cases often require radical interventions combined with multimodal therapy. Finally, the burden of treatment-related morbidity emphasizes the importance of long-term follow-up and multidisciplinary care involving urologists, oncologists, radiotherapists, and supportive care specialists.

### 3.2. Implications for Healthcare Systems

Beyond individual patient management, these findings also have implications for the healthcare system. Delayed diagnosis and advanced-stage presentation highlight the need for improved awareness among patients and primary care providers to facilitate earlier referral and timely intervention [33]. Access to specialized tertiary care centers is particularly important for complex cases requiring multidisciplinary expertise. In addition, structured referral pathways and standardized management protocols may contribute to more consistent care and improved outcomes. From a broader public health perspective, preventive strategies, including HPV vaccination programs and patient education, may help reduce disease burden over time [34].

### 3.3. Novelty and Contribution of the Study

This study should be regarded as a pilot study, providing preliminary yet clinically meaningful insights into the molecular features of penile SCC in a real-world tertiary care setting.

Despite the limited number of cases, this study integrates detailed clinical presentation, surgical management, long-term outcomes, and immunohistochemical profiling, providing a comprehensive perspective that is uncommon in small case series.

A key contribution of this retrospective study is its exploration of the relationship between HPV-associated and HPV-independent molecular profiles and their potential correlation with clinical outcomes. In our case series, we observed that p16-positive tumors were associated with more favorable short-term surgical outcomes than p16-negative advanced disease. Given our limited cohort of three patients, these findings are clinical observations rather than generalized prognostic conclusions.

Therefore, in addition to the information already presented, our study reflected the following aspects:

- the most conservative approach (partial penectomy) and the extreme (emasculation). The article does not present a homogeneous series but covers the full spectrum of penile carcinoma surgery: from maximum functional preservation (partial penectomy at pT1a) to radical salvage surgery (emasculation with perineal urethrostomy at pT3pN3). Notably, the study shows that emasculation can provide long-term local oncological control (Recurrence-Free Survival [RFS] ~3 years and 8 months) even in a p16-negative patient with advanced invasion.
- It highlights underdiagnosis in a cN0 case using standard imaging methods. Early pelvic recurrence (within 3 months) in a patient preoperatively assessed as cN0 on contrast-enhanced CT highlights the limitations of standard imaging. As a clinical novelty, this case provides strong real-world justification for early metabolic staging (18F-FDG PET/CT) or increased vigilance (follow-up interval <3 months, early pelvic dissection) in cases presenting as p16-negative with high-grade and deep invasion.
- It encourages centers with limited real-world clinical resources (impossibility of performing PCR testing). The novelty lies in highlighting the practical utility of routine immunohistochemistry (p16/p53/Ki-67) compared with techniques such as PCR in resource-limited centers.

This study highlights distinct patterns of tumor extension, including cases with predominant involvement of the corpora cavernosa and limited glans infiltration, which may contribute to delayed diagnosis and necessitate more aggressive surgical strategies.

Furthermore, the findings reinforce the central prognostic role of lymph node involvement and illustrate the potential benefit of multimodal treatment approaches for selected patients with advanced disease.

As a retrospective case series, these observations should be interpreted with caution; however, they provide a foundation for future larger-scale investigations to validate the clinical and molecular correlations observed.

### 3.4. Study Limitations

The present study has several limitations that should be acknowledged. First, its retrospective design inherently introduces selection bias. Second, the small number of cases limits the generalizability of the findings and precludes statistical analysis. In addition, the pilot nature of this study further supports the exploratory character of the results. Finally, the absence of a standardized screening protocol may have influenced the stage at diagnosis.

Despite these limitations, the strength of this study lies in its detailed clinical characterization of distinct disease presentations and their correlation with therapeutic strategies and outcomes. By integrating these observations with the current literature, this work provides a practical, clinically relevant perspective on the management of penile carcinoma.

A methodological limitation of this study is its e exclusive reliance on p16 INK4a immunohistochemistry as a surrogate marker for HPV infection. Although p16 is widely accepted in clinical practice, it does not equate to direct viral identification. This series did not test for HPV DNA via PCR or E6/E7 mRNA transcripts.

Finally, greater emphasis should be placed on early detection strategies, including public health initiatives, patient education, and expanded HPV vaccination programs. Future studies exploring the impact of early referral pathways and multidisciplinary management in specialized centers may significantly improve survival and quality of life for patients with penile carcinoma.

## 4. Conclusions

Penile carcinoma, although rare, remains a clinically challenging malignancy, with prognosis largely determined by the stage at diagnosis and the extent of lymph node involvement. The present case series highlights the direct relationship between tumor burden, nodal status, and clinical outcomes, emphasizing the critical importance of early detection and accurate staging in guiding therapeutic decision-making.

The incorporation of immunohistochemical markers, such as p16, may provide valuable insight into tumor heterogeneity and could improve risk stratification in clinical practice.

Penile carcinoma requires early detection, accurate clinical and nodal staging, and individualized surgical treatment. Organ-sparing surgery should be pursued whenever oncologically feasible, whereas advanced disease requires aggressive resection and multimodal therapy. Although p16 status serves as a useful surrogate marker, multi-center prospective studies incorporating direct viral testing and advanced PET/CT staging are warranted to further refine personalized treatment protocols.

What distinguishes this case series is its detailed documentation from an Eastern European tertiary referral center, illustrating real-world clinical decision-making when balancing guideline adherence against individual patient comorbidities, delayed presentation, and adverse histopathological risk factors.

Beyond oncological outcomes, the long-term impact on functional status and quality of life remains substantial, underscoring the importance of multidisciplinary care and prolonged follow-up. Increased awareness, timely diagnosis, and preventive strategies, including HPV vaccination, are essential to improving survival and quality of life for patients with penile carcinoma. Ultimately, optimizing outcomes in penile carcinoma depends not only on surgical expertise but also on early clinical recognition, integration of molecular insights, and timely referral to specialized centers.

This retrospective case series study may support the future integration of molecular profiling into routine clinical decision-making in penile carcinoma.

## Figures and Tables

**Table 1:** Summary of clinical characteristics, treatment, and outcomes of the included cases.

Case	Age	Comorbidities	Pathological Stage	Primary Surgical Intervention	Lymphadenectomy Extent	P16/HPV Status	Outcome/Follow-Up
1	67	Heart failure, hypertension, gastric ulcer surgery	pT2pN2	Partial penectomy	Bilateral superficial & deep inguinal LND+ extended right ilio-obturator LND (at 3 months)	HPV-independent molecular profile (p16−, p53−, p63+, Ki-67 low	Death at 31 months (pelvic recurrence at 3 months)
2	53	GERD, hiatal hernia	pT3pN3	Total emasculation + perineal urethrostomy	Bilateral superficial & deep inguinal LNDlteal	HPV-independent molecular profile (p16−, Ki-67 high)	Recurrence-free survival (RFS) ~3 years 8 months
3	73	Hypertension, CHF	pT1aN0	Partial penectomy	Bilateral superficial inguinal LND	HPV-associated molecular profile (p16+, Ki-67 moderate)	3-year Disease-Free Survival (documented follow-up through Dec 2023)

## Data Availability

The original contributions presented in this study are included in the article. Further inquiries can be directed to the corresponding authors.

## Abbreviations

The following abbreviations are used in this manuscript:

HPV	Human Papillomavirus
SCC	Squamous Cell Carcinoma
CT	Computed Tomography
EAU	European Association of Urology
GERD	Gastroesophageal Reflux Disease
RT	Radiotherapy
IHC	Immunohistochemistry
RFS	Recurrence-Free Survival

## References

[1] Compérat E., Grass G.D., Spiess P.E. (2025). Penile Cancer: Epidemiology, Pathology and Carcinogenesis. Primer on Urology.

[2] Douglawi A., Masterson T.A. (2017). Updates on the epidemiology and risk factors for penile cancer. Transl. Androl. Urol..

[3] Li Y., Liu F., Cai Q., Deng L., Ouyang Q., Zhang X.H.-F., Zheng J. (2025). Invasion and metastasis in cancer: Molecular insights and therapeutic targets. Signal Transduct. Target. Ther..

[4] Praça M.S.L., Sousa F.T.R.D., Cândido E.B., Lamaita R.M., Wender M.C.O., Silva Filho A.L. (2024). Beyond the diagnosis: Gender disparities in the social and emotional impact of cancer. Rev. Assoc. Médica Bras..

[5] Brăila A.D., Poalelungi C.-V., Albu C.-C., Damian C.M., Dȋră L.M., Bănățeanu A.-M., Bogdan-Andreescu C.F. (2025). The relationship between cervicovaginal infection, human papillomavirus infection and cervical intraepithelial neoplasia in Romanian women. Diseases.

[6] Zhang Y., Qiu K., Ren J., Zhao Y., Cheng P. (2025). Roles of human papillomavirus in cancers: Oncogenic mechanisms and clinical use. Signal Transduct. Target. Ther..

[7] Shetty M., Adiga D.S.A. (2024). Study of expression of P16 in premalignant and malignant lesions of penis and their significance. Iran. J. Pathol..

[8] Amicuzi U., Grillo M., Stizzo M., Olivetta M., Tammaro S., Napolitano L., Reccia P., De Luca L., Rubinacci A., Della Rosa G. (2024). Exploring the multifactorial landscape of penile cancer: A comprehensive analysis of risk factors. Diagnostics.

[9] Engelsgjerd J.S., Leslie S.W., LaGrange C.A. (2024). Penile cancer and penile intraepithelial neoplasia. StatPearls [Internet].

[10] Richter S., Ruether J.D., Wood L., Canil C., Moretto P., Venner P., Gingerich J., Emmenegger U., Eisen A., Zalewski P. (2013). Management of carcinoma of the penis: Consensus statement from the Canadian Association of Genitourinary Medical Oncologists (CAGMO). Can. Urol. Assoc. J..

[11] Bloom J.B., Stern M., Patel N.H., Zhang M., Phillips J.L. (2018). Detection of lymph node metastases in penile cancer. Transl. Androl. Urol..

[12] Srinivas V., Morse M., Herr H., Sogani P., Whitmore W. (1987). Penile cancer: Relation of extent of nodal metastasis to survival. J. Urol..

[13] Stecca C.E., Alt M., Jiang D.M., Chung P., Crook J.M., Kulkarni G.S., Sridhar S.S. (2021). Recent advances in the management of penile cancer: A contemporary review of the literature. Oncol. Ther..

[14] Sosnowski R., Kuligowski M., Kuczkiewicz O., Moskal K., Wolski J.K., Bjurlin M.A., Wysock J.S., Pęczkowski P., Protzel C., Demkow T. (2016). Primary penile cancer organ sparing treatment. Cent. Eur. J. Urol..

[15] Eskenazi F., Medina L.G., Soto Suarez R., Fumero L., Lusinchi Delfino A.C., Patel K., Tobias Machado M., Lee R., Sotelo R. (2025). Optimizing Inguinal Lymph Node Dissection for Penile Cancer: A Pathway to Improve Outcomes and Complications—A Narrative Review. Complications.

[16] Medina L.G., Hatoum F., Paesano N., Zemp L., Grass D., Morse B., Dhillon J., Chahoud J., Pettaway C., Spiess P.E. (2026). Multidisciplinary Care of Patients with Penile Cancer. Am. Soc. Clin. Oncol. Educ. Book.

[17] Christyani G., Carswell M., Qin S., Kim W. (2024). An overview of advances in rare cancer diagnosis and treatment. Int. J. Mol. Sci..

[18] Fitzgerald R.C., Antoniou A.C., Fruk L., Rosenfeld N. (2022). The future of early cancer detection. Nat. Med..

[19] Mititelu R., Mitoi A., Mazilu C., Jinga M., Radu F.I., Bucurica A., Mititelu T., Bucurica S. (2024). Advancements in hepatocellular carcinoma management: The role of 18F-FDG PET-CT in diagnosing portal vein tumor thrombosis. Nucl. Med. Commun..

[20] Baba S.K., Alblooshi S.S.E., Yaqoob R., Behl S., Al Saleem M., Rakha E.A., Malik F., Singh M., Macha M.A., Akhtar M.K. (2025). Human papilloma virus (HPV) mediated cancers: An insightful update. J. Transl. Med..

[21] Menon S., Moch H., Berney D., Cree I., Srigley J., Tsuzuki T., Compérat E., Hartmann A., Netto G., Rubin M.A. (2023). WHO 2022 classification of penile and scrotal cancers: Updates and evolution. Histopathology.

[22] Parza K., Mustasam A., Ionescu F., Paravathaneni M., Sandstrom R., Safa H., Grass G.D., Johnstone P.A., Eschrich S.A., Chadha J. (2023). The prognostic role of human papillomavirus and p16 status in penile squamous cell carcinoma—A systematic review. Cancers.

[23] Sun L., Zhang L., Krigman H.R., Hagemann I.S. (2018). p16 immunohistochemistry is not always required for accurate diagnosis of grade 2 squamous intraepithelial lesions. J. Low. Genit. Tract. Dis..

[24] Bogdan-Andreescu C.F., Albu Ș.-D., Slăvescu D.A., Bubulac L., Tudor V., Botoacă O., Bănățeanu A.-M., Cadar E., Albu C.-C. (2026). Periodontitis-induced immune reprogramming: Implications for cancer immunotherapy response. Biomedicines.

[25] Petrescu I., Condrea C., Alexandru A., Dumitrescu D., Simion G., Severin E., Albu C., Albu D. (2010). Diagnosis and treatment protocols of cutaneous melanoma: Latest approach 2010. Chirurgia.

[26] Zekan D.S., Dahman A., Hajiran A.J., Luchey A.M., Chahoud J., Spiess P.E. (2021). Prognostic predictors of lymph node metastasis in penile cancer: A systematic review. Int. Braz. J. Urol..

[27] Zhu Y., Ye D.-W. (2012). Lymph node metastases and prognosis in penile cancer. Chin. J. Cancer Res..

[28] Hakenberg O.W., Dräger D.L., Erbersdobler A., Naumann C.M., Jünemann K.-P., Protzel C. (2018). The diagnosis and treatment of penile cancer. Dtsch. Ärzteblatt Int..

[29] Coddington N.D., Redger K.D., Higuchi T.T. (2021). Surgical principles of penile cancer for penectomy and inguinal lymph node dissection: A narrative review. AME Med. J..

[30] Vreeburg M.T., Donswijk M.L., Albersen M., Parnham A., Ayres B., Protzel C., Pettaway C., Spiess P.E., Brouwer O.R. (2024). New EAU/ASCO guideline recommendations on sentinel node biopsy for penile cancer and remaining challenges from a nuclear medicine perspective. Eur. J. Nucl. Med. Mol. Imaging.

[31] Andreșanu A., Gîngu C., Georgescu D.E., Oliță M.R., Dobra M.A., Mirvald C., Obrișcă B., Eftimie M.-A., Sinescu I. (2026). Prognostic Factors of Survival in Patients with Surgically Treated Penile Squamous Cell Carcinoma: A Retrospective Cohort Analysis. Cancers.

[32] Chipollini J., Garcia-Castaneda J., Harb-De la Rosa A., Cheriyan S., Azizi M., Spiess P.E. (2019). Important surgical concepts and techniques in inguinal lymph node dissection. Curr. Opin. Urol..

[33] Janes W.I., Henley J., Andrews M., Organ M., Johnston P. (2023). A quality assurance review of penile cancer diagnostic delays and stage at presentation during the COVID-19 pandemic. Can. Urol. Assoc. J..

[34] Shabbir M., Barod R., Hegarty P.K., Minhas S. (2013). Primary prevention and vaccination for penile cancer. Ther. Adv. Urol..

